# High-performance liquid chromatographic analysis of water-soluble vitamins in tablets with automatic continuous-flow sample preparation

**DOI:** 10.1155/S1463924683000243

**Published:** 1983

**Authors:** S. C. Coverly

**Affiliations:** Technicon Instruments Company Ltd Hamilton Close Hampshire Basingstoke UK


Journal of Automatic Chemistry, Volume 5, Number 2 (April–June 1983), pages 89–93

### High-performance liquid chromatographic analysis of water-soluble vitamins in tablets with automatic continuous-flow sample preparation

S. C. Coverly

*Technicon Instruments Company Ltd, Hamilton Close, Basingstoke, Hampshire, UK*

### Introduction

Water-soluble vitamins in multi-vitamin preparations have been analysed by ion-pair chromatography using alkyl- and amino-bonded phase-column packings [1, 2 and 3]. However, the efficiency of high-performance liquid chromatography (HPLC) as an analytical technique in terms of analysis time can often be fully realized only where rapid sample preparation techniques are available. Ideally this should involve automated sample preparation directly coupled to the chromatograph. The use of continuous-flow techniques to perform the sample preparation in an automatic HPLC system has been described for the analysis of fat-soluble vitamins [4 and 5], and for drugs in blood serum [6 and 7]. The potential application of the technique to water-soluble vitamins has been outlined in a review by Burns [8] of systems for automated pre- and post-column reactions. This article describes a system which analyses nicotinamide, thiamin, pyridoxine and riboflavin in pharmaceutical products at a rate of five to 10 samples/h.

### Materials and methods

### *Apparatus*

The FAST-LC system used in this method (figure 1) comprises the following modules: SPS II solid sampler fitted with a rapid sample kit, auxiliary control kit, dual-speed homogenizer, diluent heater and homogenizer heater; pump III; analytical cartridge fitted with a 37°C heating bath and a 58 cm dialyser; HPLC pump; HPLC cartridge fitted with a six-port pneumatically-actuated injection valve; variable-wavelength UV detector; FAST-LC programmer; and single pen recorder (Technicon Instruments Company Ltd, Hamilton Close, Basingstoke, Hampshire, UK).

### *Reagents*

All chemicals are of analytical grade except where specified. Sample diluent and extraction solvent: 1 litre contains 7·0 g potassium dihydrogen orthophosphate. Dialysis recipient solution: 1 litre contains 1·36 g potassium orthophosphate, 0·7 g orthophosphoric acid, 0·34 g sodium heptanesulphonate and 0·1 ml triethylamine. HPLC mobile phase: 1 litre contains 40 ml tetrahydrofuran (UV grade), 0·34 g sodium heptanesulphonate, 1·36 g potassium dihydrogen orthophosphate, 0·7 ml orthophosphoric acid and 0·1 ml triethylamine. Its pH is 2·5 at 20°C.

*FAST-LC-8 column* (167 B183 01): a 150 × 4·6 mm column packed with 5 $\mu$m particles of porous silica bonded with dimethyloctylchlorosilane.

*Calibrators:* the vitamin standards used were manufactured by Roche; the internal standard, NN′diethyl nicotinamide, was obtained from the Sigma Chemical Company, Poole, Dorset, UK. A daily working standard solution is prepared such that 5 ml contains the nominal quantity per tablet of each vitamin to be measured. When preparing this solution the nicotinamide and riboflavin are added to 20 ml water. Sufficient 2 M sodium hydroxide solution is added to dissolve the riboflavin, taking care not to add an excess, which causes riboflavin to decompose, after which the other vitamins are added and the solution is diluted to 50 ml with water. The solution is stored in the dark and is stable for 10 h. 5 ml of this standard solution is measured into a sample cup for calibration. A 10% solution of the internal standard is prepared daily and a volume containing approximately as much internal standard as there is riboflavin in the product is measured into a hard gelatin capsule. One of these capsules is then placed in each sample cup.

### *Samples*

The system was used with nine different multi-vitamin and mineral products—hard and soft gelatin capsules and sugar-coated tablets—with different vitamin contents: nicotinamide 0·5–200 mg, riboflavin 0·15–5 mg, thiamin 0·1–50 mg, pyridoxine 0·05–5 mg.

### System operation

### *Sample treatment*

Samples, standards and internal standards (if used) are loaded into the sample cups.

The following operations are carried out automatically after the introduction of a sample. A pre-set volume of diluent, between 30 and 120 ml, pre-heated to 70°C by passing through a glass coil in an oil bath, is added to the homogenizer. After a short period of rotation at 500 rpm, to break the product coarsely, the homogenizer rotor runs at 2900 rpm for up to 3·5 min to dissolve the vitamins. Following a short settling period a 5 ml aliquot of the homogenizer contents is drawn into a holding coil on the sampler; it is then slowly aspirated into the continuous-flow manifold. If the sample nicotinamide content is greater than 50 mg the solution is pumped through a dilution loop to bring the nicotinamide concentration to below 0·5 $gl^{-1}$.

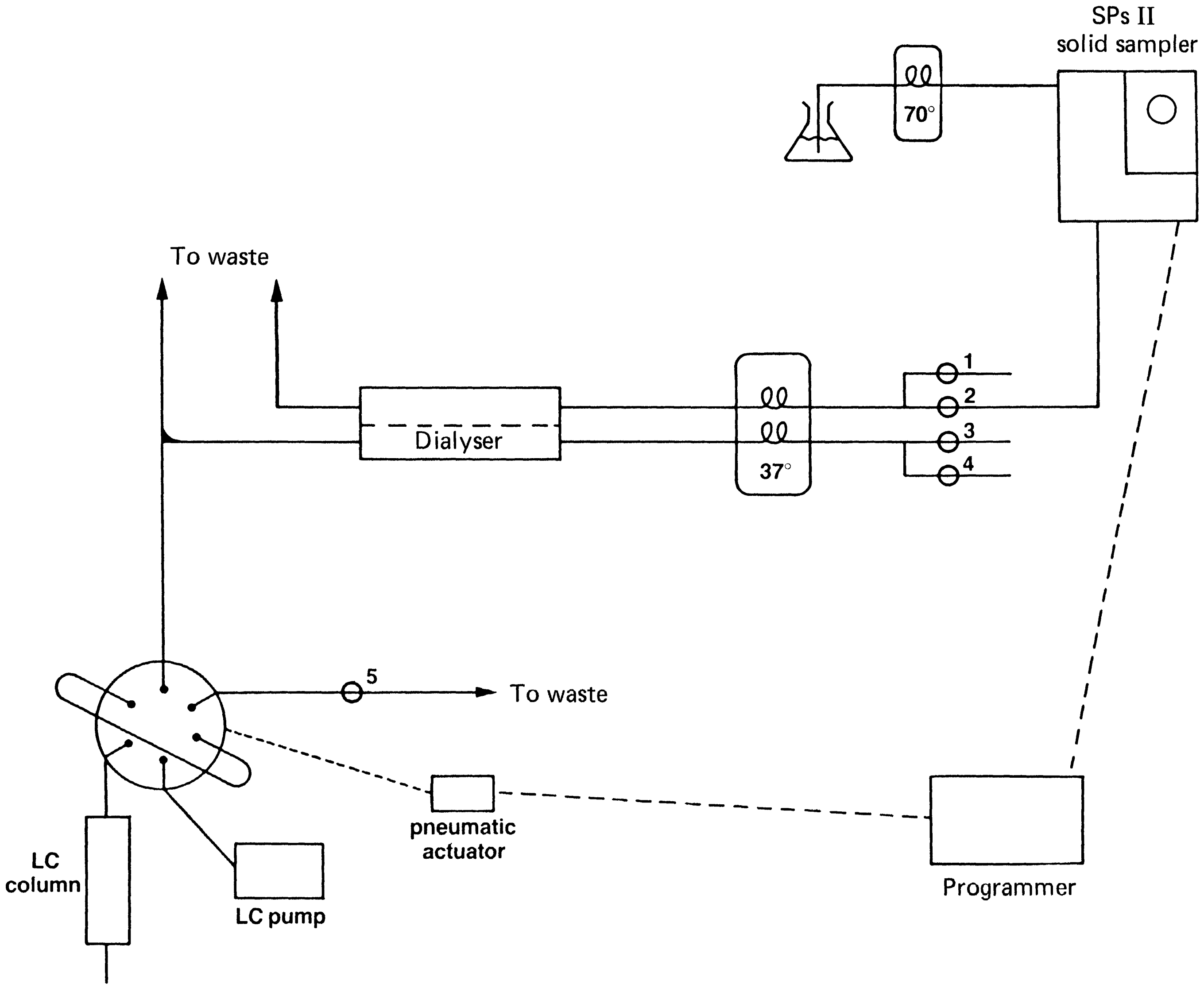

*Figure 1. Flow diagram of FAST-LC B-vitamins system. Peristaltic pump tube flow rates, ml/min. Where 1 = air 0·05, 2 = sample 0·4, 3 = recipient 0·32, 4 = air 0·05, 5 = loop pull-through 0·1. Rapid-sample pump tubes are not shown.*

It is debubbled, resegmented with a regular air injection, and pumped through a short coil in a 37°C heating bath, and then into the dialyser. An air-segmented recipient stream passes through a second coil in the 37°C heating bath before entering the dialyser—heating the streams entering the dialyser reduces the sensitivity of dialysis recovery to changes in ambient temperature. In order to reduce the elasticity of the slow-flowing air-segmented stream the frequency of air injection is reduced from the standard 2 s interval to 4 s. After leaving the dialyser the recipient stream is debubbled and flows through the sample loop of the injection valve.

### *System timing*

The sample solution from the holding coil enters the manifold over a period of 4 min. Due to dispersion, the sample band has widened to 5·5 min by the time it reaches the injection valve, and the 'steady state' plateau of maximum concentration lasts for 2·5 min. The sample loop on the valve is opened to receive the samplè shortly after the beginning of the steady-state period, and closed, injecting the sample onto the column, near the end of this period. The flow rate of the liquid through the sample loop during filling, controlled by the peristaltic pump, is 0·1 ml/min.

The timing of the operation of the injection valve and the sampler, together with automatic attenuation of the detector and changes to the LC pump flow if required, is controlled by the programmer. The necessary timing of the valve operation relative to the start of the sampling cycle is determined by phasing the system, either empirically or with the use of a detector fitted with a suitable flow-cell in place of the injection valve.

### *Sampling rate*

The sampling rate is set by the slower of the two functions of the system: sample preparation and chromatography. In order to minimize variations in the system response it is desirable to achieve a steady-state concentration of sample in the injection loop both at the moment of injection and for a period before and after the nominal injection time; otherwise, variations in the system lag time will affect the proportion of the sample which is injected. It is also desirable that there is no carry-over from one sample to the next.

Experiments showed that a 6 min cycle was necessary to give a 2·5 min period of steady-state concentration in the injection loop. While this may seem excessive in terms of AutoAnalyzer practice, if the injection loop volume is 50 μl the time taken to fill it three times over (a commonly accepted condition for complete purging) is about 1·5 min. The 30 s margin each side allows for variation in lag time due to changes in the pump tube flow rates. 6 min also represents the minimum time for a complete chromatogram when the HPLC flow rate is limited to 2 ml/min in order to maximize the resolution. Some products contained an unidentified constituent ($k = 10$) which necessitated a cycle time of 12 min. In this latter case the LC pump flow rate was increased to 4 ml/min after the elution of thiamin.

### *Sensitivity*

The sensitivity may be adjusted by the volume of extracting solvent, the dilution before dialysis and the injection volume. The upper limit is set by the linearity of the detector response to nicotinamide, which is both the highest concentration vitamin and the earliest eluted; the lower limit by the maximum injection volume which gives an acceptable peak shape, the detector wavelength and sensitivity, and the resolution of the vitamins from interferences. Experiments with injection volumes showed that the shape of the nicotinamide peak was unacceptably broad at volumes greater than 100 μl. The peak shapes of the later eluting vitamins were, however, acceptable at this volume.

The extracting solvent is set at its upper limit by the capacity of the homogenizer, and at its lower limit by the acceptable concentration of solids in the homogenized sample or by the sum of the homogenizer dead volume (25 ml) and the sample volume. The choice of detector wavelength has been discussed by Walker *et al.* [2], and 272 nm was used.

### *Start-up procedure*

Operation of the system is begun by turning on the heating baths for the sample diluent and dialyser temperature stabilization and starting the peristaltic and LC pumps. The diluent volume, cycle time and LC pump flow rate are set as previously determined for the product being analysed. The injection time will have been determined during the last phasing of the instrument. When the temperature and hydraulic conditions have reached equilibrium (about 30 min) two empty cups are run on the sampler to allow the homogenizer to reach its working temperature, followed by calibrators and samples.

### Results

Chromatograms from two products are shown in figures 2 and 3. The results were calculated from the manual measurement of the peak height of each vitamin (relative to that of the internal standard, if used), taking into account the volume of the standard solution. The system response was linear over the range of sample levels for each vitamin stated previously ($r \geqslant 0{\cdot}998$), and single-point calibration was used in routine analysis. The coefficient of variation on aqueous standards ($N = 18$) was: nicotinamide 2·2%, pyridoxine 2·0%, riboflavin 2·0%, thiamin 2·4%, using the internal standard; and 1·6%, 2·0%, 3·6% and 2·2% respectively for results calculated without reference to the internal standard. The carry-over was less than 0·5% for each vitamin and for the internal standard, using a 6 min cycle, and results were not corrected for carry-over.

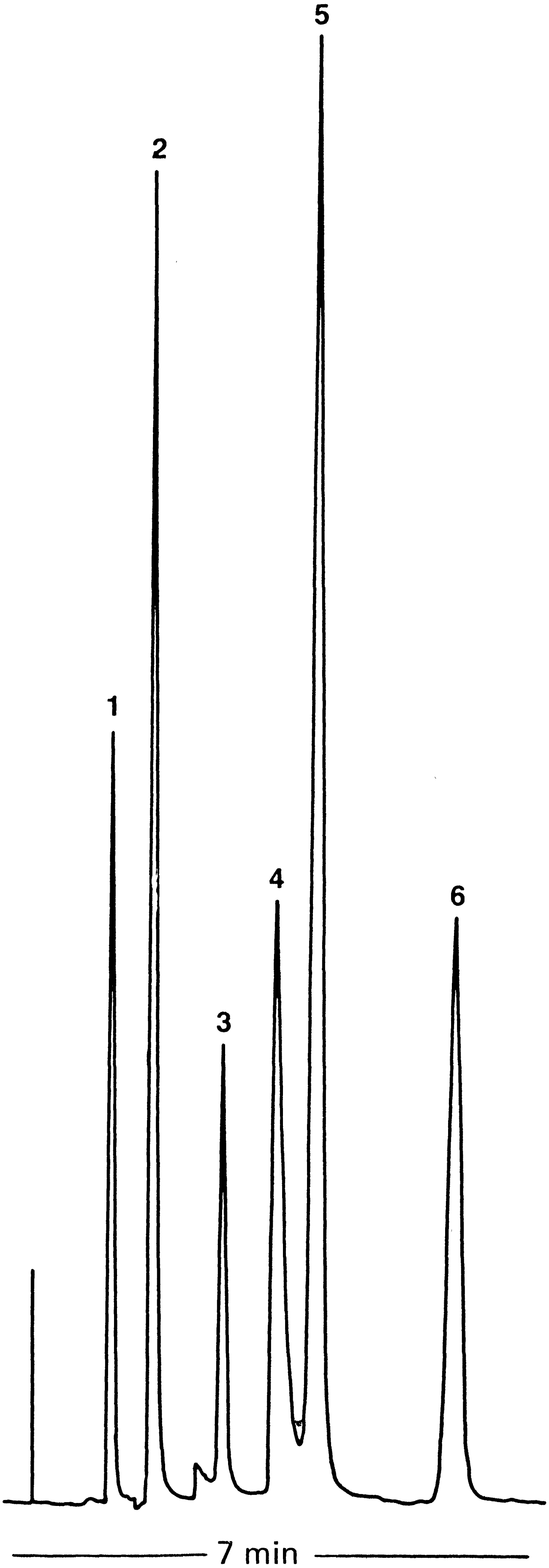

*Figure 2. Multi-vitamin tablet, 272 nm. Where 1 = ascorbic acid (not quantified), 0·64 AUFS; 2 = nicotinamide, 50 mg, 0·64 AUFS; 3 = pyridoxine, 2·5 mg, 0·04 AUFS; 4 = internal standard, 2·5 mg, 0·04 AUFS; 5 = riboflavin, 2·5 mg, 0·04 AUFS; 6 = thiamin, 2·5 mg, 0·04 AUFS.*

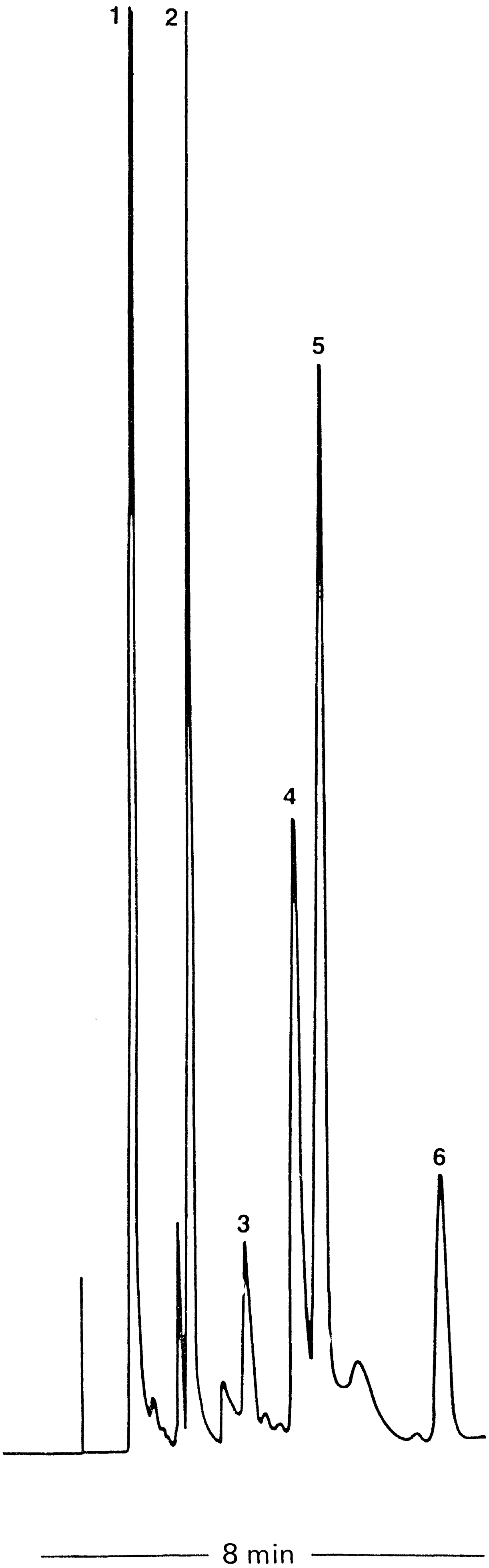

The precision and accuracy for tablet analysis were determined from spiked placebo recovery experiments. For the example shown in this article, a FAST-LC system was run on five consecutive days using a pseudo-randomized tray protocol based on the description of Daniel [9].

For this study calibrators were placed after every nine samples, which consisted of vitamin-free placebos from a micro-pelleted formulation with the four vitamins to be measured added at 33%, 66% and 100% of the calibrator concentrations. The amounts of each vitamin are typical of commonly used preparations. The diluent volume was 85 ml, the injection volume was 50 $\mu$l and the LC flow rate 1·5 ml/min. The detector attenuation (automatically scaled) was 0·16 AUFS for nicotinamide and 0·04 AUFS for later peaks. The time per sample was 8 min. As seen in table 1, the day-to-day precision (coefficient of variation) is between 1·8 and 5·8% for the four vitamins at three levels. The recovery for each vitamin in this study is also shown in table 1.

Two defects were noted when the complete system was first operated. First, thiamin appeared as two peaks, with base-line separation, on some samples. This was remedied by the inclusion of the ion-pairing agent in the recipient stream. As observed by Walker *et al.* [2], products containing iron showed a broad peak in the middle of the chromatogram and low recoveries compared to standards without iron. This was overcome by adding a quantity of disodium ethylenediamino-tetraacetic acid (EDTA), equal to the quantity of iron in the sample, to the sample diluent. While the use of EDTA results in a usable chromatogram, the interference is not completely eliminated and it is necessary to include iron with the standards. Due to the constant extraction conditions, and time between extraction and injection for both samples and standards, the degradation of thiamin noted by Walker *et al.* [2] does not adversely affect the results from this system.

### Discussion

The data in table 1 demonstrate the system's performance with samples of different content in the same run. When only one product is being analysed, with the extraction and LC conditions optimized for the levels concerned, the day-to-day coefficients of variation are similar to those for the highest concentration in this study, namely from 2·0% for thiamin to 2·7% for riboflavin. Kirchmeier and Upton [3] reported reproducibilities between 0·5% and 3·0% on batches of four replicates on five products; the sample preparation consisted of 10 min dissolution in an ultrasonic bath, followed by dilution to volume and filtration; the LC analysis time was 35 min. Walker *et al.* [2] analysed 10 products with reproducibilities over a three-month period of between 0·6% and 2·0%; the samples were shaken for 60 min and then filtered; the LC analysis time was 30 min, and an average of 16 samples were analysed per day. Allowing one hour for start-up and shut-down and assuming one calibrator for every nine samples, the system described in this paper analyses 36 to 72 samples per 9 h day.

The system has successfully analysed products intended for human consumption of the types and concentrations described;

*Figure 3. Multivitamin and mineral tablet (25 components), 272 nm. Where 1 = ascorbic acid (not quantified), 0·32 AUFS; 2 = nicotinamide, 20 mg, 0·32 AUFS; 3 = pyridoxine, 1 mg, 0·04 AUFS; 4 = internal standard, 2 mg, 0·04 AUFS; 5 = riboflavin, 2 mg, 0·04 AUFS; and 6 = thiamine, 2 mg, 0·04 AUFS.*

*Table 1. Recovery and precision data for about 90 assays—six were performed at each level for five days.*

| Vitamin | Weighed-in quantity (mg) | Mean (mg) | Recovery (%) | Day-to-day precision (SD,mg) | (CV%) | Within-run precision (SD,mg) | (CV%) |
|---|---|---|---|---|---|---|---|
| Nicotinamide | 15·00 | 14·94 | 99·7 | 0·39 | 2·6 | 0·35 | 2·3 |
| | 9·90 | 10·02 | 103·3 | 0·29 | 2·9 | 0·26 | 2·6 |
| | 4·95 | 5·21 | 105·2 | 0·26 | 5·3 | 0·21 | 4·3 |
| Pyridoxine, B6 | 2·50 | 2·51 | 100·2 | 0·060 | 2·4 | 0·058 | 2·3 |
| | 1·65 | 1·65 | 100·0 | 0·036 | 2·2 | 0·033 | 2·0 |
| | 0·825 | 0·82 | 99·4 | 0·048 | 5·8 | 0·039 | 4·7 |
| Riboflavin, B2 | 0·50 | 0·496 | 99·2 | 0·014 | 2·7 | 0·013 | 2·5 |
| | 0·33 | 0·339 | 102·9 | 0·007 | 2·1 | 0·006 | 1·9 |
| | 0·165 | 0·173 | 105·2 | 0·006 | 3·6 | 0·005 | 3·3 |
| Thiamin, B1 | 2·00 | 1·99 | 99·3 | 0·040 | 2·0 | 0·038 | 1·9 |
| | 1·32 | 1·34 | 101·2 | 0·024 | 1·8 | 0·022 | 1·7 |
| | 0·66 | 0·64 | 97·6 | 0·024 | 3·7 | 0·024 | 3·6 |

it was not suitable for a veterinary product containing meat and bone meal due to blockage of the manifold tubing with meat fibres. The system can be converted to the analysis of fat-soluble vitamins [4 and 5]: for this the manifold assembly carrying the dialyser and heating bath is exchanged for one-holding fittings for solvent extraction, after which the system is re-equilibrated with the reagents for the new analysis, and the programmer timing is reset.

### Conclusion

A fully automated method for the analysis of water-soluble vitamins in tablets and capsules has been developed. Linearity, recovery and reproducibility are similar to previously reported manual LC methods; the analysis rate is increased and errors due to the decomposition of thiamin after extraction from mineral-containing formulations are reduced. The system can be easily adapted to the analysis of fat-soluble vitamins.

### Acknowledgements

I thank Dr I. D. Cockshott and Dr R. Macrae for their advice on the preparation of the manuscript.

### References

1. Wills, R. B. H., Shaw, C. G. and Day, W. R., *Journal of Chromatographic Science*, **15** (1977), 262.
2. Walker, M. C., Carpenter, B. E. and Cooper, E. L., *Journal of Pharmaceutical Science*, **70** (1981), 99.
3. Kirchmeier, R. L. and Upton, R. P., *Journal of Pharmaceutical Science*, **67** (1978), 1444.
4. Dolan, J. W., Gant, J. R., Giese, R. W. and Karger, B. L., *Journal of Chromatographic Science*, **16** (1978), 616.
5. Abdou, H. M., Russo-Alesi, F. M. and Fernandez, V., *Pharmaceutical Technology* (March 1981).
6. Dolan, J. W., van der Wal, S., Bannister, S. J. and Snyder, L. R., *Clinical Chemistry*, **26** (1980), 871.
7. Bannister, S. J., van der Wal, S., Dolan, J. W. and Snyder, L. R., *Clinical Chemistry*, **27** (1981), 849.
8. Burns, D. A., *Pharmaceutical Technology* (March 1981).
9. Daniel, C., *Journal of Qualitative Technology*, **7** (1975), 103.

### PHILIPS X-RAY CONFERENCE IN DURHAM

Delegates to the 1983 Philips conference on X-ray analysis will stay in the 11th-century Durham Castle; the meeting takes place at University College. The full technical programme will deal with all aspects of X-ray fluorescence spectrometry. Preliminaries consist of registration on Monday 19 September in the afternoon, followed by a sherry reception on the evening. Technical sessions take place from Tuesday 20 September to Friday 23 September, with Wednesday afternoon set aside for scientific discussion or sight-seeing. The price of £135·00 includes conference fee, accommodation, meals and visits, but non-residential delegates will also be accepted.

*Applications for conference circulars and offers of papers should be made to Maureen Courtney at Pye Unicam, York Street, Cambridge CB1 2PX, UK. Tel.: 0223 358866.*

